# Dynamic compression of dense oxide (Gd_3_Ga_5_O_12_) from 0.4 to 2.6 TPa: Universal Hugoniot of fluid metals

**DOI:** 10.1038/srep26000

**Published:** 2016-05-19

**Authors:** N. Ozaki, W. J. Nellis, T. Mashimo, M. Ramzan, R. Ahuja, T. Kaewmaraya, T. Kimura, M. Knudson, K. Miyanishi, Y. Sakawa, T. Sano, R. Kodama

**Affiliations:** 1 Graduate School of Engineering, Osaka University, Suita, Osaka 565-0871, Japan; 2 Photon Pioneers Center, Osaka University, Suita, Osaka 565-0871, Japan; 3 Department of Physics, Harvard University, Cambridge, Massachusetts 02138, USA; 4 Shock Wave and Condensed Matter Research Center, Kumamoto University, Kumamoto 860-8555, Japan; 5 Condensed Matter Theory Group, Department of Physics and Astronomy, Box 516, Uppsala University, SE-751 20, Uppsala, Sweden; 6 Applied Materials Physics, Department of Materials Science and Engineering, KTH Royal Institute of Technology, SE-100 44, Stockholm, Sweden; 7 Geodynamics Research Center, Ehime University, Ehime 790-8577, Japan; 8 Sandia National Laboratories, Albuquerque, New Mexico 87185-1181, USA; 9 Institute for Shock Physics, Washington State University, Pullman, WA 99164-2816, USA; 10 Institute of Laser Engineering, Osaka University, Suita, Osaka 565-0871, Japan; 11 Institute for Academic Initiatives, Osaka University, Suita, Osaka 565-0871, Japan

## Abstract

Materials at high pressures and temperatures are of great current interest for warm dense matter physics, planetary sciences, and inertial fusion energy research. Shock-compression equation-of-state data and optical reflectivities of the fluid dense oxide, Gd_3_Ga_5_O_12_ (GGG), were measured at extremely high pressures up to 2.6 TPa (26 Mbar) generated by high-power laser irradiation and magnetically-driven hypervelocity impacts. Above 0.75 TPa, the GGG Hugoniot data approach/reach a universal linear line of fluid metals, and the optical reflectivity most likely reaches a constant value indicating that GGG undergoes a crossover from fluid semiconductor to poor metal with minimum metallic conductivity (MMC). These results suggest that most fluid compounds, *e.g.,* strong planetary oxides, reach a common state on the universal Hugoniot of fluid metals (UHFM) with MMC at sufficiently extreme pressures and temperatures. The systematic behaviors of warm dense fluid would be useful benchmarks for developing theoretical equation-of-state and transport models in the warm dense matter regime in determining computational predictions.

At the end of the twentieth century, novel materials were being made systematically through multiple shock (quasi-isentropic) compression. Hydrogen, nitrogen, and oxygen completed crossovers from fluid semiconductor to fluid metal at extreme pressures of 100–140 GPa (1.0–1.4 Mbar) and temperatures of a few thousand kelvins^1^,^2^,^3^. Metallization of fluid H occurs at ~3,000 K and 0.64 mol H/cm^3^, essentially the density predicted by Wigner and Huntington, 0.62 mol H/cm^3 ^4. At that H density, the Fermi temperature is *T*_*F*_ ≈ 220,000 K and the degeneracy factor is *T*/*T*_*F*_ ≈ 0.014. Electrical conductivities of those ultra-condensed degenerate fluids reach a common value, *i.e.*, the minimum metallic conductivity (MMC), that results from strong scattering of the conduction electrons with mean-free path lengths comparable to interatomic distances^2^,^5^.

Substantially higher pressures and temperatures are achieved and measured with single-shock compression than with multiple-shock compression^6^,^7^. The locus of states achieved by a series of shock jumps, each starting from a given initial density to a sequence of ever-increasing shock pressures, is called a Rankine-Hugoniot curve or simply a Hugoniot. Hugoniots of elemental metals, such as Al, Cu, Fe, and Mo, have been calculated at shock pressures even up to 10^4^ TPa^8^. These theoretical Hugoniots have complex shapes in pressure-volume space, which derive from partial ionization of the atomic shells at pressures above 10 TPa. Experimental Hugoniot data at ultrahigh shock pressures have been measured by several investigators using shock waves generated by nuclear and chemical explosives, by a giant laser and by magnetic-field driven hypervelocity impact for comparison with theoretical calculations. Predicted shell-structure effects on Hugoniots at ultrahigh shock pressures and temperatures have yet to be observed definitively; one possible reason is the large errors in measured extreme shock pressures and volumes.

In contrast, we found another systematic behavior of fluid metals from the Hugoniot data for the typical metals in the range between sub-TPa and ~10 TPa. This pressure range is well below that where shell-structure effects become significant for the shape of the Hugoniot curve. The Al, Cu, Fe, and Mo Hugoniot data show, surprisingly, that all lie on a common straight line in shock velocity (*U*_*s*_) - particle velocity (*u*_*p*_) space, as a preliminary study found^9^. No significant deviations of those data from the common linear fit has been observed. We here call this common fit the universal Hugoniot of fluid metals (UHFM).

The temperature of Al shock-compressed to ~1 TPa, for example, reaches ~62,000 K. Basically, fluid metals under multi-TPa shock pressures can approach or attain warm dense matter (WDM) states in which the potential energy of electron-ion interactions is comparable to the kinetic energies of electrons (*i.e., T*/*T*_*F*_ ≈ 1). The challenge with fluid metals in the warm dense matter regime is to understand the correlated-electron-ion physics of partially ionized fluids, and to develop the equation-of-state (EOS) and transport models. Matter at such high *P*-*T* conditions is of increasing scientific interest for several reasons: (i) understanding the physics of warm dense matter, (ii) understanding the interiors of giant exoplanets now being discovered, and (iii) the need for alternative sources of commercial energy. EOS and transport properties at extreme conditions are needed to model the interior structure and dynamics of giant planets and to design inertial fusion energy experiments driven by a giant pulsed laser and by magnetic-field driven hypervelocity impacts. Systematic behaviors of warm dense fluid would be useful as benchmarks for developing theoretical EOS and transport properties for use in computational simulations.

For these reasons, we have measured the Hugoniot and optical reflectivity of a strong, dense oxide or insulating compound to extend the systematics to other type of materials. Specifically, Gd_3_Ga_5_O_12_ (GGG) has been chosen for our experimental and computational investigations because at shock pressures in the range 200–250 GPa^10^ it melts on its Hugoniot and is therefore a suitable oxide fluid to study at the higher shock pressures that are explored. Because of the high initial density of GGG (*ρ*_0_ = 7.1 g/cm^3^) and the consequent high shock impedance, strong shock compression of GGG allows us to create a dense oxide fluid even at conditions corresponding to the deep interiors of giant planets and exoplanets.

## Ultrahigh-pressure experiments

**Figure 1:**
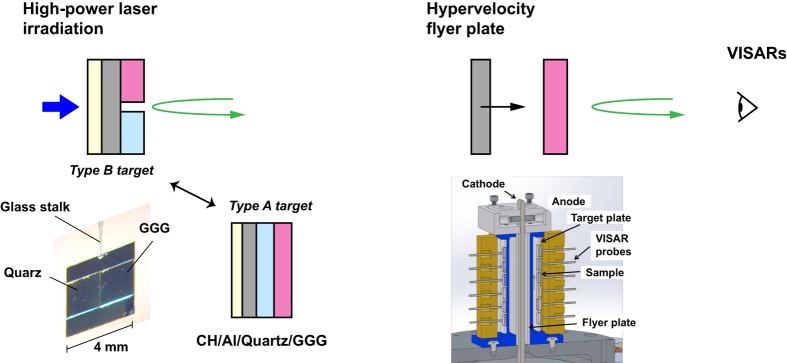
Configurations on laser (left) and Z-accelerator experiment (right). Two types of target assemblies are used in the laser experiments. The type A target assembly consists of layers of an Al shine-through shield, polypropylene (CH) ablator, Al pusher, *α*-quartz (SiO_2_) reference, and GGG sample. The laser beams first hit the CH ablator spray-coated with a 0.1 *μ*m-thick Al layer to block direct laser shine-through. With type B targets, both SiO_2_ and GGG plates are assembled side-by-side on the Al pusher as shown in the target photograph. Both sides of the GGG specimens are polished and the VISAR observation side was anti-reflection-coated for a 532-nm VISAR probe light. In the Z-accelerator experiments, Al flyer plate is propelled by a large magnetic field, and then the GGG sample is impacted by the flyer plate accelerated to a hypervelocity of ~24–33 km/s.

Ultrahigh-pressure shock compression experiments for GGG were performed on the GEKKO XII laser facility at the Institute of Laser Engineering (ILE), Osaka University^11^ and on the Z-accelerator facility at the Sandia National Laboratory^12^. Figure 1 shows the schematics of the configurations of both experiments. As we show later, GGG Hugoniot data from these experiments using completely different techniques show very good agreement. The detail of the Z-accelerator experiments is briefly described in the section Methods because this technique is more conventional like a typical dynamic compression using light gas gun than laser shock compression.

For the laser shock experiments, we used high-intensity pulsed laser beams at 351-nm wavelength. The temporal shape of the laser pulse was approximately square with a 2.5-ns duration (full width at half maximum) and rise and fall times of ~100 ps each. The focal-spot diameter was typically 600 *μ*m with a flat-top distribution resulting in a planar shock front of more than 400 *μ*m in diameter^13^,^14^. A laser energy of up to ~520 J was deposited onto the focal spot, resulting in an averaged laser irradiation intensity of up to ~7.4 × 10^13 ^W/cm^2^. With this level of laser intensity, the high shock impedance of GGG allows us to generate multi-TPa pressures.

Plate-shaped single-crystal GGG samples were used for the experiments. The laser beams first hit a 15-*μ*m or 30-*μ*m thick CH plastic ablator to prevent preheating of the target by minimizing X-rays generated in the coronal plasma. GGG Hugoniots were determined using the references of Al and/or *α*-quartz based on the impedance matching technique^15^. Shock velocities of GGG and quartz were measured with two line-imaging velocity interferometers (line-VISARs)^16^. The line-VISARs were also used to measure shock reflectivity *R*.

## Results and Discussion

**Figure 2:**
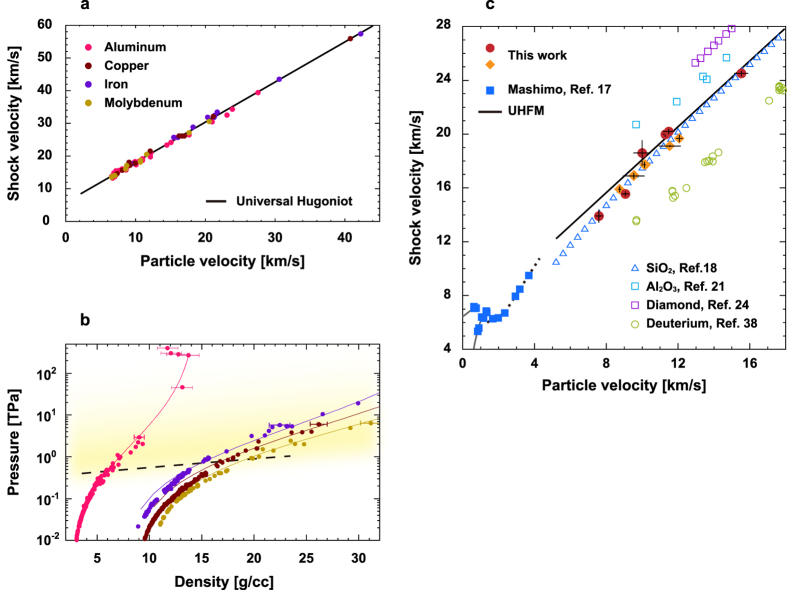
Universal Hugoniot of fluid metals. Universal Hugoniot of fluid metals (**a**) in the *U*_*s*_ vs. *u*_*p*_ (solid line) and (**b**) in the *P* vs. *ρ* from previously published data for Al, Cu, Fe, and Mo. In Fig. 2(a), shock pressures corresponding to shock velocities near 60 km/s are ~20 TPa. (**c**) Hugoniot data of GGG and the UHFM line. Red circles and orange diamonds are our laser data and *Z* data, respectively. Solid blue squares signify data from gas-gun experiments^17^. The solid straight line above *u*_*p*_ = 5 km/s is the UHFM line^9^, and the open triangles from the Hugoniot of *α*-quartz (SiO_2_) from the *Z* experiments^18^. The GGG data, as well as the SiO_2_, are reasonably consistent with the UHFM. D_2_ (open green circles)^38^, Al_2_O_3_ (open blue squares)^21^, and diamond (open purple squares)^24^ are typical exceptions.

**Universal Hugoniot of fluid metals.** In Fig. 2(a) we show experimental evidence of the UHFM obtained from published Hugoniot data for typical metals at very high shock pressures above ~0.5 TPa. When the Hugoniot data of Al, Cu, Fe, and Mo are plotted as *U*_*s*_(*u*_*p*_) they have a fair common linear fit *U*_*s*_ = *c*_0_ + *su*_*p*_, with *c*_0_ = 5.8 km/s and *s* = 1.2 for 5 km/s < *u*_*p*_ < 45 km/s, within a few percent in *U*_*s*_ at a given *u*_*p*_^6^,^7^,^9^.

As seen in Fig. 2(b), the UHFM models reasonably approximate the shape of Hugoniot curves of those metals even above 10 TPa within the experimental data uncertainties. The asymptote to the UHFM line of the Hugoniot data for each metal suggests a crossover to warm dense fluid metal. Below the crossover point pressures (indicated by the dashed line), the Hugoniot data significantly deviate from the UHFM line except for Al. This exception could be due to the melting temperature of Al being considerably lower than for the other three metals. At ultrahigh *P*-*T* (≳10 TPa and ≳10^6^ K) along the Hugoniot, for which the ionization of inner-shell electrons becomes remarkable, the UHFM might be inconsistent. However, this has not yet been confirmed experimentally.

Figure 2(c) shows that GGG Hugoniot data approach the UHFM line above 0.75 TPa and are reasonably consistent with the line above ~1 TPa; the present data are summarized in Table 1. This implies that strong shocks rapidly break the chemical bonds of GGG above the melting point and the subsequent Gd-Ga-O mixture at sufficiently high shock pressures and temperatures attains the common state of metallic fluids. As described later the shock temperature of GGG at ~0.8 TPa is ~22,000 K. This temperature is much higher than the melting temperature of GGG at ambient (~5,000 K)^10^, and therefore at sufficiently high shock pressures GGG would become a fluid system on the UHFM.

**Figure 3:**
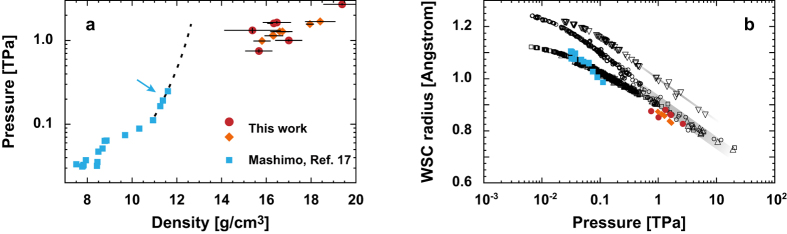
Pressure - density and WSC radius - pressure along the Hugoniot of GGG. (**a**) Hugoniot data of GGG plotted as *P* vs. *ρ*. A large change of volume occurs probably above the melting point of GGG along the Hugoniot. Red circles and orange diamonds are laser data and *Z* data, respectively. Blue squares signify data from ref. 17. The dotted line is the extrapolation of the gas gun data in the incompressible semiconducting phase above *u*_*p*_ = 2.3 km/s. GGG crosses over to a metal above *u*_*p*_ = 4 km/s (Fig. 2). (**b**) Shock pressure dependence of the Wigner-Seitz cell radii of GGG and typical metals (Al: open circles, Cu: squares, Fe: triangles, Mo: inverse triangles, and Be: diamonds). Pressure dependence of WSC radii of these materials becomes similar at sufficiently high shock pressures and temperatures.

Figure 3(a) shows the relationship between pressure and density for GGG along the Hugoniot. Compared with the the previous gas-gun data extrapolated up to 0.3 TPa (dotted line)^17^, our GGG data show a significant increase in density with increasing pressure. The dotted line in this figure corresponds to the dotted line shown in Fig. 2(c). The experimental densities at ~1 TPa are approximately 30% higher than predicted from the extrapolation. GGG melts at ~0.21 TPa shock pressure and ~5,000 K shock temperature along the Hugoniot as mentioned in the previous study^10^. The shock melt region along the Hugoniot, indicated by the blue arrow in the figure, signifies that a transition to a highly compressible fluid has occurred extremely rapidly above the shock melting temperature. This is fully consistent with the fact that the GGG Hugoniot data approach the UHFM above ~0.75 TPa and almost reach the line above ~1 TPa.

Figure 3(b) shows the Wigner-Seitz cell (WSC) radius for GGG along the Hugoniot compared with those of typical metals. WSC radius *r*_*ws*_ is here approximated by


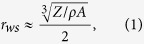


where the *ρ, Z*, and *A* are density along the Hugoniot, atomic weight, and Avogadro’s number, respectively, the equivalent to the averaged interatomic distance of a material. At low pressures below 0.1 TPa each metal exhibits its characteristic WSC radius and its differentiation *dr*_*ws*_/*dP*, reflecting interatomic correlations and/or chemical bond strength. In contrast, above shock pressures of ~0.5 TPa, metals lose these characteristics; *e.g., dr*_*ws*_/*dP* becomes very similar to the slope of the gray lines drawn in the figure. Strongly shock-compressed metals most likely cross over to a common fluid state with a similar interatomic correlation or strength. Our GGG data follow this systematic behavior of fluid metals. GGG decomposes to its constituent elements Gd, Ga, and O, by strong shock compression, and simultaneously that their interatomic distances become similar in magnitude as those in typical fluid metals lying on the UHFM.

As shown in Fig. 2(c), the quartz Hugoniot also displays characteristics of a UHFM above sufficiently high shock pressures^18^. The fitting curve of the quartz Hugoniot can be approximated to be *U*_*s*_ = 5.89 + 1.22*u*_*p*_ with a residual error of more than 0.999 above ~850 GPa pressure corresponding to ~14 km/s particle velocity. At sufficiently high shock pressures and temperatures, Hugoniots of even strong oxides or insulating compounds above melting approach the common line for UHFM.

The shock pressure at which a material reaches or approaches the UHFM depends on the strength of a material at atmospheric pressure. Liquid deuterium (D_2_) has negligible strength at atmospheric pressure. At shock pressures in the range from 70 to 110 GPa, the Hugoniot of liquid D_2_ is *U*_*s*_ = *c*_0_ + *su*_*p*_, with *c*_0_ = 1.70 km/s and *s* = 1.22^19^, whereas for the UHFM *c*_0_ = 5.90 km/s and *s* = 1.22. The slopes *s* are same for these two cases. However, *c*_0_ = 5.9 km/s is typical of simple fluid metals, whereas *c*_0_ = 1.70 km/s is typical of a low-density highly compressible metallic fluid, displacing the D_2_ Hugoniot significantly below UHFM.

Shock-compression curves of the strongest crystals are also displaced above the UHFM because shock pressures that cause melting are so high that Hugoniot data are affected substantially by the solid phase and relatively weakly by the fluid phase in comparison to most solids. The Hugoniot of Al_2_O_3_ up to 0.34 TPa is approximately linear in *U*_*s*_(*u*_*p*_) with *c*_0_ = 8.74 km/s and *s* = 0.96^20^. The *c*_0_ of Al_2_O_3_ is much larger than that of the UHFM. This high *c*_0_ value of Al_2_O_3_ arises from its initial high velocities of sound and incompressibilities, which is expected to cause high deviations from the UHMF. In contrast, the *s* of Al_2_O_3_ is slightly smaller than that of the UHFM. At higher shock pressures (≳750 GPa), at which shock temperatures increases substantially with increasing shock pressure, the slope *s* increases substantially for Al_2_O_3_ from 0.96 to 1.08^21^,^22^. The *c*_0_ and *s* of diamond also show a similar behavior. At shock pressures in the range from 0.6 to 1.9 TPa, *U*_*s*_(*u*_*p*_) is approximately linear with *c*_0_ = 1.2 km/s and *s* = 1.01^23^, with relatively small variations around the complex melt region between 0.6 and 1.1 TPa^24^,^25^.

To summarize here briefly, the systematics in fluid metals were extendable to warm dense fluid states of strong oxides or insulating compounds initially with localized chemical bonds. The systematic behaviors of the shock compression EOS data (Hugoniot) allow us to categorize warm dense fluid metals into three broad types. The first is a very soft, highly compressible fluid compressed starting from, *e.g.*, a molecular system with initially negligible strength. The second is a very strong, much less compressible liquid with a significant interatomic correlation, strength, and/or chemical bonding even at TPa shock pressures. Finally, the third is a fluid metal on the UHFM, which most materials reach.

### Reflectivity measurements

**Figure 4:**
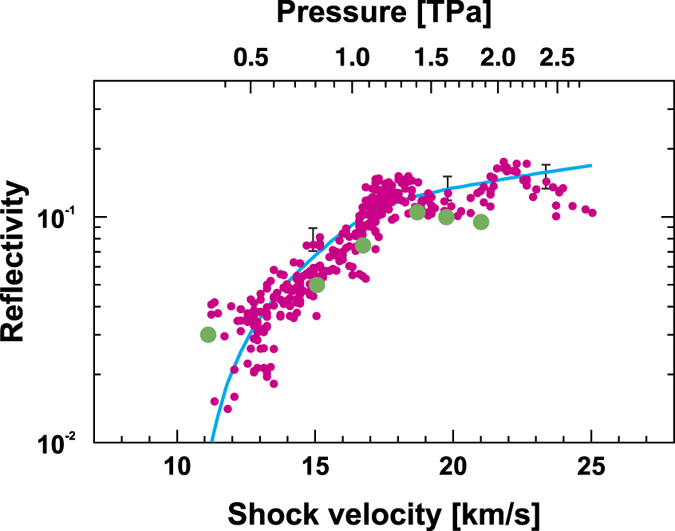
Experimental and theoretical optical reflectivities. We confirmed a very good agreement between experiments and calculations. The measured GGG optical reflectivities at 532 nm (pink dots) vs. shock velocity (bottom axis) and vs. shock pressure (top axis). Blue line is a smooth fit of the experimental data for the guide to eyes. The experimental reflectivity increases exponentially with pressure and most likely saturates above ~1.4 TPa shock pressures. Green solid circles are reflectivities calculated theoretically as described in text at pressures calculated at corresponding densities using *c*_0_ and *s* coefficients of the UHFM.

We measured optical reflectivity *R* along the GGG Hugoniot up to ~2.6 TPa with the line-VISARs using the smoothly decaying shock technique^25^. Absolute *R* was determined by cross-checking using the two methods of comparing shock reflectivity, *i.e.*, from the surface of the Al pusher and from the shocked quartz^13^,^26^,^27^. Measured *R* in the range 0.015 to 0.15 at shock velocities corresponding to shock pressures from ~0.4 to ~2.6 TPa, are plotted in Fig. 4. The reflectivity increases exponentially with shock velocity and pressure and then most likely saturates above ~18 km/s and ~1.4 TPa. This behavior of reflectivity is consistent with the behavior of the GGG *U*_*s*_-*u*_*p*_ data that reach the UHFM line at the velocity and pressure. A similar trend in optical reflectivity was observed by a different group^28^.

In the previous study, at shock pressures from 110 to 250 GPa, *dc* electrical conductivities of GGG, measured from 10^−3^ to 10^3^ S/m with metallic contact probes show that GGG has a band gap of 3.1 eV^17^. Thermal equilibrium in a thin shock front in GGG is established by optical absorption and scattering at shock pressures from 120 to 130 GPa^10^. Extrapolation of the measured GGG electrical conductivities to shock pressure above 0.25 TPa implies GGG achieves MMC (~10^5 ^S/m) of a strong-scattering fluid at higher shock pressures, a pressure beyond the upper range achievable with a two-stage light-gas gun. This is consistent with the present experiments in that optical reflectivity begins to be observed above ~0.4 TPa.

In the present decaying shock experiment, we simultaneously measured the emission intensities to deduce the temperature of a shocked sample using a streaked self-emission diagnostics^13^. From this measurement, GGG shock temperatures corresponding to shock pressures of ~0.8 and 2.5 TPa are ~22,000 and 45,000 K, respectively. Once shock pressures and temperatures become sufficiently high as those for GGG, the electronic structures are expected to cross over to those of fluid metals with the EOS coinciding with the UHFM. Other strong oxides or insulating compounds are expected to undergo similar crossovers in the fluid metal phase, at which chemical bonds cross over to electronic energy bands. Because of disorder in the fluid, electron scattering is strong with mean-free path comparable to interatomic distances. Because strong scattering is weakly sensitive to particular atoms undergoing scattering, electrical conductivity is expected to be weakly sensitive to chemical composition, and therefore to be MMC. The crossover to a fluid with MMC on the UHFM is probably a general effect in fluid metals of materials at sufficiently high shock pressures and temperatures, although there are a few notable exceptions as described above.

Theoretical calculations show that very strong, electrically insulating Al_2_O_3_ undergoes a crossover to a poor metal with MMC on the Hugoniot. The shock-compression curve and associated electrical conductivities of Al_2_O_3_ have been calculated for shock pressures up to 1.5 TPa. Those calculations indicate that Al_2_O_3_ melts at *P* ~ 0.5 TPa and 10,000 K, at which electrical conductivity becomes significant. This shock melting pressure is more than twice the pressure of GGG, and additionally the Al_2_O_3_ electrical conductivity increases up to 2 × 10^5^ S/m at 0.9 TPa^29^. These results imply that shock compression to sufficiently higher pressures transforms the strongest oxides or compounds from an electrical insulator to a fluid metal with MMC and, furthermore, probably pushes the Hugoniot towards the UHFM.

### *Ab-initio* molecular dynamics simulations

**Figure 5:**
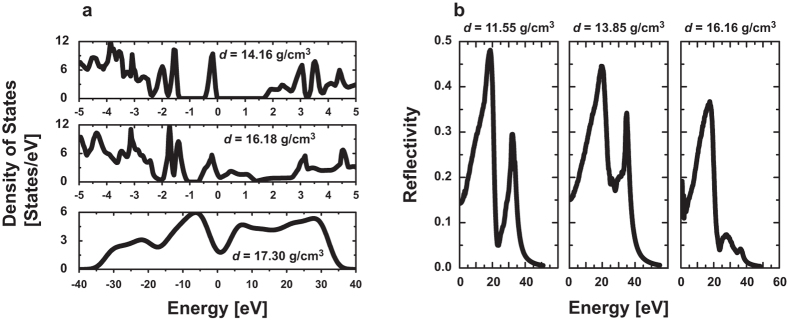
DOS and reflectivity of shock-compressed GGG. (**a**) Density of states (DOS) at *ρ* = 14.16 g/cm^3^ (top), at 16.18 g/cm^3^ (middle), and 17.30 g/cm^3^ (bottom). (**b**) Calculated reflectivities vs. photon energy for three densities of amorphous GGG. The photon wavelength in all reflectivity experiments was 532 nm. The optical reflectivity of crystalline GGG is 0.11, which is subtracted from each calculated reflectivity to obtain the increase in reflectivity caused by shock compression.

To confirm the experimental reflectivity, we performed *ab-initio* molecular dynamics simulations, finding a good agreement between experiment and theory (Fig. 4). Electronic structures, densities of electronic states (DOS), and optical reflectivities at wavelength 532 nm were calculated for six shock densities greater than the GGG crystal density at ambient conditions. Pressures corresponding to those chosen densities were calculated using the UHFM. The values of these physical quantities are listed in Table 2. The calculated DOS is illustrated for three densities in Fig. 5(a). The system is semiconductor with a band gap of ~1.6 eV at *d* = 14.16 g/cm^3^. However, a small density of states at the Fermi level can be observed at *d* = 16.18 g/cm^3^, which become prominent at *d* = 17.3 g/cm^3^, as no finite band gap is observed at these densities. The calculated energy-dependent optical reflectivities are illustrated in Fig. 5(b). A sufficient increase in reflectivity at *d* = 16.16 g/cm^3^ is indicative of band gap closure (metallic behavior) for this material.

## Methods

### Velocity interferometry observations in the laser experiments

The VISAR system used has two different velocity sensitivities (velocity-per-fringe, VPF) to resolve 2*π*-phase shift ambiguities. The sensitivities are 4.122 and 14.69 km/s/fringe taking the refractive index of SiO_2_ into account. The VISAR probe is an injection-seeded, *Q*-switched YAG laser, operated at a wavelength of 532 nm with a pulse duration of ~10 ns at FWHM. We measured both shock velocities of quartz and GGG using this VISAR system. Our analysis of the VISAR records show uncertainties in the measured *U*_*s*_ to be typically 2% or less.

### Impedance matching Hugoniot determination

For the laser experiments, we used two types of target (Fig. 1). For type A targets, quartz and GGG *U*_*s*_ just before and after the shock arrival at the Qz/GGG interface were used for determining the GGG Hugoniot point. The shocked quartz state was obtained from the measured quartz *U*_*s*_ and the known quartz Hugoniot^18^,^30^. We used the fitting curve of the quartz Hugoniot data derived from the hypervelocity impact experiments; 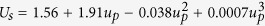. Then, GGG *u*_*p*_ was determined by matching the shock impedances of GGG and quartz using the measured GGG *U*_*s*_ and the quartz reshock curve. To derive this off-Hugoniot curve, we used the Mie-Grüneisen EOS theory and the Grüneisen gamma parameter for quartz, depending on shock velocity^31^. The GGG pressure and density are determined exactly from the *U*_*s*_ and *u*_*p*_ by the Rankine-Hugoniot relations. For type B targets, the shocked Al state was inferred from the measured quartz *U*_*s*_, and the known Al Hugoniot and release curve. We used a linear fitting curve that well approximated the Al Hugoniot; *i.e., U*_*s*_ = 6.341 + 1.185*u*_*p*_^30^. We used the Mie-Gru
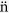
eisen EOS theory and the Gru
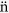
eisen gamma parameter of Al with the form *γ*_0_(*ρ*_0_/*ρ*)^32^. Then, GGG *u*_*p*_ was determined by matching shock impedances of GGG and Al using the measured GGG *U*_*s*_ and the Al reshock curve starting from the inferred Al Hugoniot state^32^.

### Hypervelocity impact experiments

Hypervelocity impact experiments were conducted at the Sandia *Z* machine, a pulsed power accelerator capable of generating ~20 MA currents, and ~10 MGauss magnetic fields in a short circuit load. The load, which is nominally 4–5 cm in each dimension, is designed to compress the cathode and explode the anode outward as flyer-plates, producing impact velocities in excess of 30 km/s^12^,^33^.

Upon discharge of the stored energy within the Marx capacitor banks, a shaped current pulse of ~300 ns duration and ~20 MA in magnitude is directed through the experimental load. The large current induces a large magnetic field, and the resulting ***J*** × ***B*** force propels the flyer-plates outward. With proper load design and temporal shaping of the current pulse, accelerations of a few tens of Gg are produced that drive the solid aluminum panels across a 3–5 mm vacuum gap, ultimately reaching impact velocities of ~24–33 km/s, depending upon the load geometry and the peak charge voltage of the accelerator.

The flyer-plates and samples were diagnosed using VISAR. As the GGG sample is transparent, the 532nm laser light could pass through the GGG and reflect off the flyer-plate surface. This arrangement enables an in-line measurement of the flyer-plate velocity from initial motion to impact. Upon impact, a ~1 TPa shock was sent through the sample. This shock was of sufficient magnitude that the shocked GGG became reflective in the visible range. The onset of reflectivity allowed for direct measurement of the shock velocity within the GGG using the VISAR diagnostic. Typical uncertainties in impact and shock velocities were of order 1% or less.

### AIMD simulations

For theoretical analysis, we used the projector augmented wave (PAW) method^34^ as implemented in the Vienna *ab initio* simulation package (VASP)^35^,^36^ to perform our density functional theory (DFT) calculations. The exchange-correlation functional was treated by the PBE variant of the generalized gradient approximation (GGA)^37^. The kinetic energy cutoff of 600 eV and 5 × 5 × 5 K-points mesh generated by the Γ method were found suitable to achieve sufficient convergence within an accuracy of 0.005 eV. The electronic self-consistent cycle was fixed at 10^−7^ eV/cell and the forces on all ions became smaller than 10^−5^ eV/Å. The standard conjugate method was used to perform the structural optimizations. *Ab initio* molecular dynamics (AIMD) simulations within the DFT framework, as implemented in VASP^35^,^36^, were performed to model and study the amorphous structures.

Using the canonical NVT (constant number, volume and temperature) approach, we heated the GGG structure from 0 to 8,000 K to establish the melting transition, at which the system lost initial memory. We used the Verlet algorithm to integrate the equation of motion. The molten states of these structures were attained by performing a MD run for 10 ps (10 × 1000 fs) with a time step of 1 fs (1 fs = 10^−15^ s) and this temperature (8,000 K) was maintained for a further 10 ps with another 10,000 MD runs. Next the mean square displacement and radial distribution function of these structures were calculated (not shown here for brevity) to analyze and confirm the molten states. After ensuring the liquid states, we rapidly cooled down these structures from 8,000 K to 300 K in 5 ps (5,000 MD runs) and finally the last snapshots of these structures were structurally optimized at 0 K to obtain fully quenched amorphous structures.

## Additional Information

**How to cite this article**: Ozaki, N. *et al*. Dynamic compression of dense oxide (Gd_3_Ga_5_O_12_) from 0.4 to 2.6 TPa: Universal Hugoniot of fluid metals. *Sci. Rep.* **6**, 26000; doi: 10.1038/srep26000 (2016).

## Figures and Tables

**Table 1:** Shock compression Hugoniot data for GGG.

	Shot number	Shock velocity (km/s)	Particle velocity (km/s)	Pressure (TPa)	Density (g/cm^3^)
Laser exp.	31813	20.19 ± 0.17	11.49 ± 0.32	1.647 ± 0.048	16.47 ± 0.63
	31822	19.98 ± 0.17	11.30 ± 0.12	1.603 ± 0.022	16.34 ± 0.29
	31829	18.59 ± 0.94	10.01 ± 0.48	1.321 ± 0.092	15.38 ± 1.26
	31836	15.57 ± 0.17	9.07 ± 0.21	1.003 ± 0.025	17.00 ± 0.60
	33120	13.90 ± 0.47	7.62 ± 0.20	0.750 ± 0.032	15.67 ± 0.81
	33565	24.51 ± 0.18	15.14 ± 0.39	2.633 ± 0.070	18.55 ± 0.80
*Z*-accelerator exp.	Z2333N	16.94 ± 0.06	9.56 ± 0.04	1.150 ± 0.005	16.30 ± 0.14
	Z2375	19.11 ± 0.06	11.55 ± 0.05	1.567 ± 0.007	17.95 ± 0.17
	Z2332	19.68 ± 0.25	12.08 ± 0.18	1.687 ± 0.025	18.41 ± 0.67
	Z2333S	17.72 ± 0.22	10.20 ± 0.08	1.283 ± 0.010	16.73 ± 0.43
	Z2345S	16.89 ± 0.21	9.54 ± 0.07	1.143 ± 0.009	16.34 ± 0.41
	Z2345N	15.90 ± 0.20	8.75 ± 0.07	0.988 ± 0.008	15.81 ± 0.38
	Z2371N	17.72 ± 0.22	10.14 ± 0.07	1.276 ± 0.010	16.61 ± 0.42

**Table 2:** 

Density (g/cm^3^)	Pressure (TPa)	Reflectivity (*R* – *R*_0_) R_0_ = 0.11
11.55	0.25	0.03
14.16	0.81	0.05
15.13	1.07	0.075
16.18	1.40	0.105
16.70	1.63	0.10
17.30	1.83	0.095

*Ab-initio* reflectivity. Shock pressures and corresponding optical reflectivities of GGG calculated with *ab-initio* molecular dynamics simulations at various densities. Pressures at various densities were calculated using the respective densities and the *c*_0_ and *s* coefficients of the UHFM.
